# Interactions of 172 plant extracts with human organic anion transporter 1 (SLC22A6) and 3 (SLC22A8): a study on herb-drug interactions

**DOI:** 10.7717/peerj.3333

**Published:** 2017-05-25

**Authors:** Hang Lu, Zhiqiang Lu, Xue Li, Gentao Li, Yilin Qiao, Robert P. Borris, Youcai Zhang

**Affiliations:** School of Pharmaceutical Science and Technology, Tianjin University, Tianjin, China

**Keywords:** Organic anion transporter, Herb-drug interactions, Plant extracts

## Abstract

**Background:**

Herb-drug interactions (HDIs) resulting from concomitant use of herbal products with clinical drugs may cause adverse reactions. Organic anion transporter 1 (OAT1) and 3 (OAT3) are highly expressed in the kidney and play a key role in the renal elimination of substrate drugs. So far, little is known about the herbal extracts that could modulate OAT1 and OAT3 activities.

**Methods:**

HEK293 cells stably expressing human OAT1 (HEK-OAT1) and OAT3 (HEK-OAT3) were established and characterized. One hundred seventy-two extracts from 37 medicinal and economic plants were prepared. An initial concentration of 5 µg/ml for each extract was used to evaluate their effects on 6-carboxylfluorescein (6-CF) uptake in HEK-OAT1 and HEK-OAT3 cells. Concentration-dependent inhibition studies were conducted for those extracts with more than 50% inhibition to OAT1 and OAT3. The extract of *Juncus effusus*, a well-known traditional Chinese medicine, was assessed for its effect on the *in vivo* pharmacokinetic parameters of furosemide, a diuretic drug which is a known substrate of both OAT1 and OAT3.

**Results:**

More than 30% of the plant extracts at the concentration of 5 µg/ml showed strong inhibitory effect on the 6-CF uptake mediated by OAT1 (61 extracts) and OAT3 (55 extracts). Among them, three extracts for OAT1 and fourteen extracts for OAT3 were identified as strong inhibitors with IC_50_ values being <5 µg/ml. *Juncus effusus* showed a strong inhibition to OAT3 *in vitro*, and markedly altered the *in vivo* pharmacokinetic parameters of furosemide in rats.

**Conclusion:**

The present study identified the potential interactions of medicinal and economic plants with human OAT1 and OAT3, which is helpful to predict and to avoid potential OAT1- and OAT3-mediated HDIs.

## Introduction

Herbal products have increasingly been incorporated into modern health care, with approximately 20% of the population taking herbal products in the United States (Bent, 2008). Consumption of herbal products, particularly when used concomitantly with conventional medications, may increase the risk of severe and potentially even fatal herb-drug interactions (HDIs) (Bilgi et al., 2010). It has been reported that about 7% of the co-users of herbal medicines and prescription medications in the United States had experienced adverse effects (Bush et al., 2007). Therefore, detailed information about possible interactions between herbal products and drugs is needed to prevent HDIs.

Organic anion transporters (human: OATs; rodent: Oats) are members of the solute carrier family SLC22A, and mediate the transport of a wide range of low molecular weight substrates (Nigam et al., 2015). OAT1 and OAT3 are almost exclusively expressed in the kidney, responsible for the renal secretion of a large variety of drugs, including angiotensin- converting-enzyme (ACE) inhibitors, diuretics, antibiotics, and antivirals (Burckhardt, 2012). Both Oat1- and Oat3-null mice are viable and fertile (Eraly et al., 2006; Sweet et al., 2002), but blood pressure was decreased about 15% only in Oat3-null mice (but not in Oat1- or Oat2-null mice) (Vallon et al., 2008). Additionally, OAT3 was shown to be required for influenza A virus replication, and an OAT3 inhibitor was shown to be effective in limiting influenza A virus infection (Perwitasari et al., 2013). Therefore, OAT1 and OAT3 are not only drug transporters, but may also be therapeutic targets for hypertension and influenza A infection.

OAT1 and OAT3 have been identified as the targets for potential HDIs. For example, components in the herbal medicine *Salviae miltiorrhizae Radix et Rhizoma* including lithospermic acid, rosmarinic acid, salvianolic acid A, salvianolic acid B and tanshinol have been shown to inhibit human OAT1 and OAT3 (Wang & Sweet, 2012). The anthraquinones in rhubarb have been identified as strong inhibitors to human OAT1 and OAT3 (Ma et al., 2014). However, few studies have been conducted to systematically investigate the interactions of herbal extracts with human OATs. In this study, we examined the effects of 172 extracts from 37 medicinal and economic plants on the function of OAT1 and OAT3, to investigate whether novel HDIs might exist. It must be noted that a number of the plants that we have studied are used as foods or beverages rather than drugs. As such, people consuming them may not realize the potential they represent for interacting with drugs or other herbal products that they are also employing as medicines.

## Materials and Methods

### Drugs and reagents

Probenecid, furosemide, warfarin and poly-D-Lysine were purchased from Sigma-Aldrich Chemical Co. (St. Louis, MO, USA). 6-Carboxyfluorescein (6-CF) was obtained from Aladdin (Shanghai, China). Dulbecco’s modified Eagle’s medium (DMEM), fetal bovine serum (FBS) and trypsin were purchased from Gibco (Gaithersburg, MD, USA). Hygromycin B was purchased from Solarbio (Beijing, China). BCA protein assay kit was purchased from Cwbio (Beijing, China). Methanol, n-hexane, dichloromethane, and n-butanol were purchased from J&K Chemical (Beijing, China). All other chemicals, unless indicated, were purchased from Sigma-Aldrich Co. (St. Louis, MO, USA).

### Plant extracts

A total of 37 medicinal and economic plants were collected by staff members from the New York Botanical Garden (Mr. Daniel Atha) and the National Herbarium of the Republic of Georgia (Dr. Manana Khutsishvili) during 2006 at various sites in the Republic of Georgia, Armenia, Azerbaijan, and the United States. Voucher specimens have been prepared and deposited in the National Herbarium of Georgia and the New York Botanical Garden. Each sample was identified by the staff of these herbaria using standard botanical methods. A list of the taxa investigated in the current study and their relative information are presented in Table S1. The detailed preparation method of plant extracts is shown in Fig. S1. Briefly, methanol extracts were prepared from either the entire plant or discrete parts of the plant. Each methanol extract was further fractionated to provide four fractions, namely n-hexane (H), dichloromethane (D), n-butanol (B) and aqueous (A). As a result, a total of 172 plant extracts were prepared, and maintained at −20 °C until use.

### Cloning and expression of human OAT1 and OAT3

HEK293 cells (bought from Invitrogen, Carlsbad, CA, USA) stably expressing OAT1 and OAT3 were established using the Flp-In expression system (Invitrogen) according to the manufacturer’s protocol as described in a previous publication (Chen et al., 2009). In brief, the cDNAs of OAT1 (GenBank accession number NM_004790) and OAT3 (GenBank accession number NM_004254) were obtained from OriGene (Rockville, MD, USA), and sub-cloned into pcDNA5/FRT (Invitrogen). They were co-transfected with pOG44 into Flp-in-293 cells. Stable cells (HEK-OAT1 and HEK-OAT3) were selected in the presence of hygromycin B (75 μg∕ml). Cells were routinely grown in DMEM containing 10% FBS, 1% streptomycin/penicillin and hygromycin B (75 μg∕ml) in a humidified incubator at 37 °C and 5˘% CO_2_. The mRNAs of OAT1 and OAT3 in three cell lines were quantified by quantitative real-time PCR. The method and primer sequences are shown in Table S2.

### Uptake assay

Cell uptake assay was performed as previously described with some modifications (Hsueh et al., 2016). Cells were seeded at a density of 7 × 10^4^ cells per well in poly-D-lysine-coated 96-well culture plates. Transport assays were performed 16-h post seeding in preheated uptake buffer (135 mM NaCl, 5 mM KCl, 2.5 mM CaCl_2_, 1.2 mM MgCl_2_, 0.8 mM MgSO_4_, 28 mM glucose, and 13 mM Hepes, pH 7.2). 6-CF was used as the fluorescent substrate for both hOAT1 and hOAT3 according to a previous study (Duan et al., 2012). The 6-CF alone or 6-CF with different inhibitors (probenecid or plant extracts) was incubated for 4 min in HEK-OAT1 cells and 10 min in HEK-OAT3 cells. Uptake was terminated by adding ice-cold phosphate-buffered saline (PBS), and quickly washed three times with ice-cold PBS. The cells in each well were lysed with 100 μl of 20 mM Tris–HCl containing 0.2% TritonX-100. An aliquot of 50 μl lysate was used to determine fluorescence using a Tecan Infinite M200 (Mannedorf, Switzerland) with excitation and emission wavelengths at 485 and 528 nm, respectively. The protein concentration of the cell lysate was determined using a BCA Protein Assay Kit (Cwbio, Beijing, China). Uptake values were standardized against protein content, and were measured in triplicate.

Uptake kinetics were characterized by measuring the uptake of increasing concentration of 6-CF (0–500 μM) in cells stably expressing hOAT1 (4 min) and hOAT3 (10 min). Michaelis constants were obtained by fitting to the Michaelis–Menten equation *V* = *V*_max_*S*∕(*K*_m_ + *S*), where *V*_max_ is the maximum transport rate, *S* is the substrate concentration and *K*_m_ is the substrate concentration reaching half-maximal uptake rate (Graphpad Prism version 5, San Diego, CA, USA).

The fluorescence of each extract in the presence or absence of 6-CF was measured to rule out detection error. Probenecid was loaded into four corners of each 96-well plate, and used as a positive control. The stock solutions of plant extracts were prepared using DMSO with a final concentration of 1 mg/ml. The initial concentration for each extract was set at 5 μg∕ml. Every well contained the same concentration of DMSO (0.5%) to eliminate the potential effect of solvent. Uptake of 6-CF (4 μM) was quantified for 4 min for OAT1 and 10 min for OAT3 in the presence or absence of plant extracts with a final concentration of 5 μg∕ml. Before the initial screening, the reproducibility of uptake experiments was evaluated. The uptake of 6-CF (4 μM) in HEK-OAT1 and HEK-OAT3 cells were measured in the absence and presence of probenecid (100 μM) in three plates with four repeated wells per uptake assay in each plate. The results demonstrated that the inhibitory effect of probenecid on OAT-mediated 6-CF uptake was reproducible (Fig. S3). Due to the large number of samples, for the initial screening study, each uptake experimental sample was measured in triplicates in the same assay. For those plant extracts showing more than 50% inhibition, the uptake experiments were repeated twice to validate the results.

Those plant extracts showing more than 50% inhibition of 6-CF uptake were selected to carry out concentration-dependent inhibition experiments. To determine IC_50_ (50% inhibitory concentration) values, three-fold serial dilutions of the tested extracts (0–5 μg∕ml) and probenecid (0–200 μM) were prepared in triplicate in a 96-well plate and mixed with an equal volume of 6-CF (4 μM). In the experiment, we found that DMSO at concentrations up to 2% had little effect on OAT-mediated 6-CF uptake (Fig. S4). Therefore, the control group contained 0.25% DMSO for the concentration-dependent inhibition experiments. After the uptake was terminated at 4 min and 10 min for OAT1 and OAT3 respectively, intracellular fluorescence was determined as above. IC_50_ values were estimated by non-linear regression analysis, using the following equation: $Y=100/((1+X \hat {} HillSlope)/({\mathrm{IC}}_{50} \hat {} HillSlope))$; where *X* is the logarithm of inhibitor concentration; *Y* is the normalized response; and HillSlope is the Hill slope factor (Graphpad Prism Version 5; Graphpad, San Diego, CA, USA).

### Pharmacokinetic studies

Pharmacokinetic study of furosemide was performed according to a previous publication (Ma et al., 2014). Male Sprague–Dawley (SD) rats (200–220 g) were obtained from Vital River Laboratories (Beijing, China), and housed in the certified animal facility at the Institute of Radiation Medicine of the Chinese Academy of Medical Sciences (CAMS, Tianjin, China). All of the experimental protocols and animal handling were performed with the approval of the Animal Use Committee at the CAMS (No. 1202). Rats were acclimated for 1 week in a temperature controlled room (23 ± 2 °C), with a relative humidity of 55 ± 10%. Food and water were supplied *ad libitum.* The rats were fasted for 12-h before the experiment. For the interaction by *Juncus effusus* extract, a dose of 100 mg/kg of this extract (dissolved in 40% PEG400) was administered to the rats by either oral gavage or tail vein injection at 5 min before administration of 10 mg/kg FS (dissolved in saline) intravenously through the tail vein. For the control group, a same volume of 40% PEG400 was administered to the rats at 5 min before the injection of a same volume of saline. About 700 μl blood was collected into heparinized tubes via the orbital venous sinus at 2, 5, 10, 15, 30, 60, 90, and 120 min. Plasma sample (250 μl) was pipetted into a microcentrifuge tube, and 1 ml acetonitrile (containing 40 μg∕ml warfarin as internal standard) was added. The mixture was vortexed and centrifuged at 20,000 rpm for 15 min. The supernatant was dried in vacuum and then was reconstituted in 200 μL mobile phase for HPLC injection.

### Quantification of furosemide by HPLC-UV

The concentrations of furosemide were quantified using a modified HPLC method (Jankowski, Skorek-Jankowska & Lamparczyk, 1997; Ma et al., 2014). Chromatographic analysis was performed on an Agilent 1260 HPLC system (Agilent Technologies, Santa Clara, CA, USA) consisting of a G1311C Quaternary pump, G1329B autosampler (0.1–100 μL), G1316A column oven (273–333 K), and G1315D Diode Array Detector (190–950 nm). The analytical column was a ZORBAX SB-C18 columm (4.6 × 150 mm, 5 µm; Agilent, Santa Clara, CA, USA) with a precolumn. The mobile phase was a mixture of acetonitrile and distilled water (69:31, v/v) with 0.3 M sodium acetate buffer (pH 5.0). The column temperature is 30 °C with a flow rate of 1 ml/min. The injection volume was 10 μl, and the detection wavelength was set at 280 nm.

### Data analysis

For the pharmacokinetics, five rats per group were used. Data with error bars represent means ± S.D. Pharmacokinetic parameters were calculated using a non-compartmental analysis by using PKSolver (Zhang et al., 2010). Data were analyzed with two-tailed unpaired Student’s *t*-test. The *p* value for statistical significance was set to be <0.05.

## Results

### Characterization of OAT1- and OAT3-overexpressing cells

**Figure 1:**
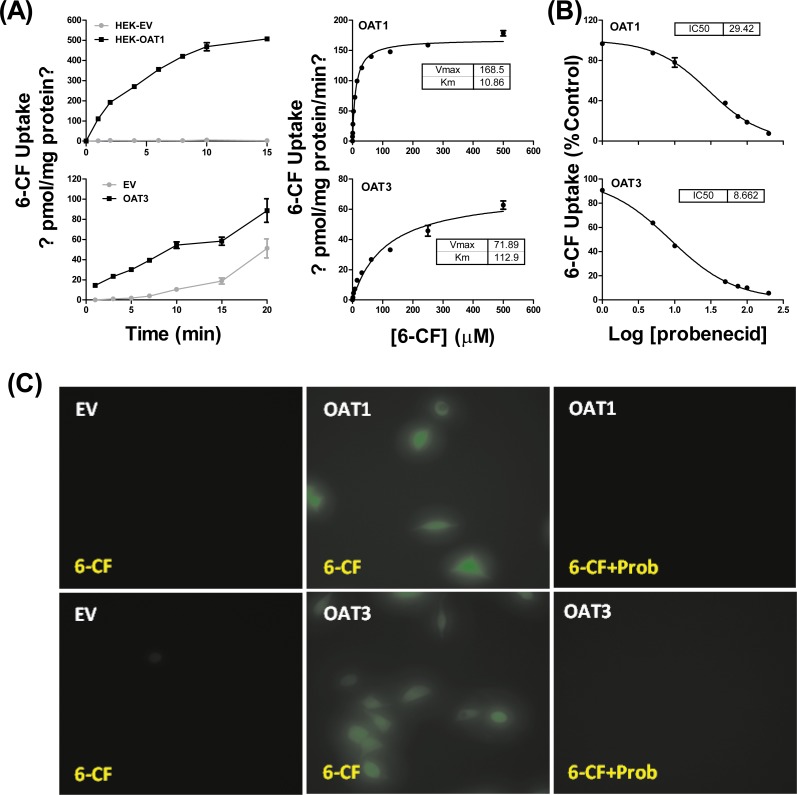
Characterization of OAT1- and OAT3-expressing HEK293 cells. (A) Time course and saturation curve of 6-CF uptake in HEK-EV, HEK-OAT1 and HEK-OAT3 cells. Means ± SD of triplicate determination (*n* = 3) were given. (B) Dose-dependent inhibition of probenecid on 6-CF uptake in HEK-OAT1 and HEK-OAT3 cells. Each data point is the mean of triplicate values (*n* = 3), and the line represents a best fit of the data using nonlinear regression analysis. (C) Fluorescence images of 6-CF uptake in the absence or presence of probenecid (30 μm) in HEK-EV, HEk-OAT1, and HEK-OAT3 cells.

HEK293 cells stably expressing OAT1 and OAT3 (HEK-OAT1 and HEK-OAT3) were validated by both mRNA expression and uptake function. As shown in Fig. S2, the mRNA expressions of OAT1 and OAT3 were almost undetectable in HEK-EV cells, whereas they are markedly increased in HEK-OAT1 and HEK-OAT3 cells, respectively. Both HEK-OAT1 and HEK-OAT3 cells exhibited a time- and concentration-dependent increase in the uptake of 6-CF (Fig. 1A). The estimated *K*_*m*_ values for the uptake of 6-CF by hOAT1 and hOAT3 were 10.86 ± 0.64 μM and 112.9 ± 14.24 μM, respectively. The *V*_max_ values of 6-CF uptake for HEK-OAT1 and HEK-OAT3 cells were 168.5 ± 2.27 and 71.89 ± 3.42 pmol/mg protein/min, respectively. Probenecid is a classic inhibitor for OAT1 and OAT3. The estimated IC_50_ value of probenecid was 29.42 μM for OAT1, and 8.662 μM for OAT3 (Fig. 1B). Fluorescence images of cells treated with 6-CF in the presence or absence of probenecid were taken using a fluorescence microscope (Nikon E100; Nikon, Tokyo, Japan). As shown in Fig. 1C, no detectable fluorescence was observed in HEK-EV cells treated with 6-CF. Both HEK-OAT1 and HEK-OAT3 cells treated with 6-CF showed a strong fluorescence, which was almost completely abolished by co-incubating probenecid. Therefore, HEK-OAT1 and HEK-OAT3 cells were successfully established and characterized by functional assay.

### Effects of 172 plant extracts on OAT1- and OAT3-mediated 6-CF transport

**Figure 2:**
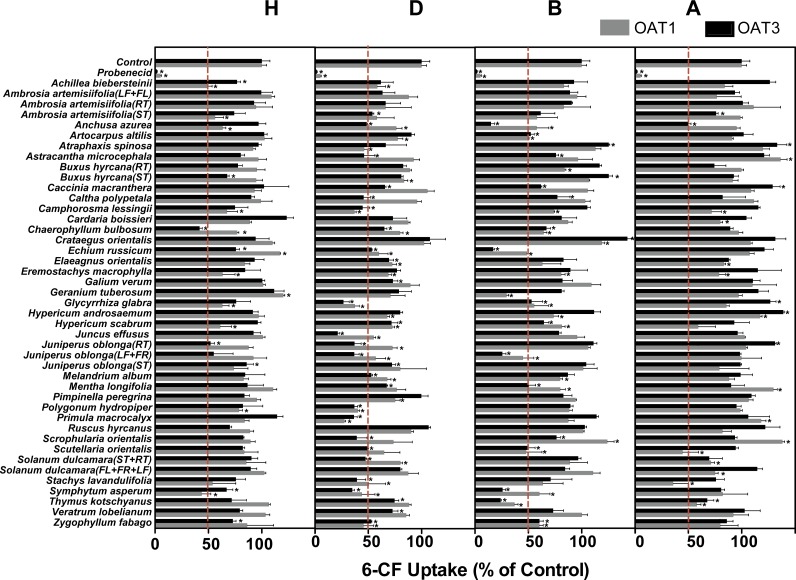
Inhibitory effects of 172 plant extracts on OAT-mediated 6-CF. Uptake of 6-CF (4μM) was measured at 37 °C for 4 min with HEK-OAT1 and 10 min with HEK-OAT3 cells in the absence or presence of the indicated plant extracts (5 μg∕ml). Probenecid (30 μM) was used as a positive control. Means ± SD of triplicate determinations (*n* = 3) were given. *P < 0.05. H, Hexane; D, Dichloromethane; B, n-Butanol; A, Aqueous.

Among the 172 plant extracts investigated in the initial screening study, 59 extracts (9H, 24D, 18B, and 10A) significantly inhibited the 6-CF uptake in HEK-OAT1 cells, whereas nine extracts (2H, 2B and 5A) significantly increased the OAT1-mediated uptake of 6-CF (Fig. 2 and Table 1). Similarly, 55 extracts (8H, 28D, 16B, and 3A) significantly inhibited the OAT3-mediated uptake of 6-CF, whereas seven extracts (2B and 5A) increased the 6-CF uptake in HEK-OAT3 cells.

**Table 1:** Effects of plant extracts on OAT-mediated 6-CF (Numbers stand for the percentage of control. Red means decrease. Green means increase.).

Botanical name	Plant part used	H		D		B		A	
		OAT1	OAT3	OAT1	OAT3	OAT1	OAT3	OAT1	OAT3
*Achillea biebersteinii*	Entire plant	50%	77%	59%	62%	83%	93%	84%	126%
*Ambrosia artemisiifolia*	Leaf and flower	109%	99%	88%	63%	96%	89%	77%	94%
	Root	95%	93%	66%	66%	83%	91%	126%	101%
	Stem	56%	74%	58%	54%	58%	61%	99%	76%
*Anchusa azurea*	Entire plant	63%	97%	76%	49%	58%	15%	95%	50%
*Artocarpus altilis*	Leaf	103%	102%	78%	91%	49%	52%	91%	102%
*Atraphaxis spinosa*	Entire plant	92%	97%	46%	66%	113%	125%	120%	133%
*Astracantha microcephala*	Entire plant	97%	80%	92%	46%	97%	76%	137%	121%
*Buxus hyrcana*	Root	95%	78%	89%	83%	84%	117%	100%	74%
	Stem	95%	68%	84%	81%	107%	125%	92%	93%
*Caccinia macranthera*	Entire plant	93%	102%	106%	66%	106%	62%	109%	129%
*Caltha polypetala*	Entire plant	99%	90%	96%	46%	103%	77%	111%	82%
*Camphorosma lessingii*	Entire plant	67%	75%	37%	45%	74%	105%	72%	116%
*Cardaria boissieri*	Entire plant	89%	123%	88%	73%	87%	81%	80%	104%
*Chaerophyllum bulbosum*	Entire plant	77%	42%	80%	65%	64%	67%	97%	89%
*Crataegus orientalis*	Leaf	110%	94%	102%	108%	119%	143%	108%	132%
*Echium russicum*	Entire plant	117%	76%	60%	53%	49%	17%	108%	121%
*Elaeagnus orientalis*	Entire plant	84%	93%	72%	69%	63%	83%	84%	88%
*Eremostachys macrophylla*	Entire plant	63%	84%	69%	77%	81%	89%	79%	115%
*Galium verum*	Entire plant	101%	101%	90%	73%	109%	82%	110%	111%
*Geranium tuberosum*	Entire plant	120%	112%	71%	79%	30%	81%	97%	116%
*Glycyrrhiza glabra*	Entire plant	63%	76%	38%	27%	55%	53%	86%	127%
*Hypericum and rosaemum*	Entire plant	97%	92%	68%	80%	74%	111%	117%	139%
*Hypericum scabrum*	Entire plant	61%	96%	73%	72%	81%	65%	59%	93%
*Juncus effusus*	Stem	101%	92%	55%	21%	96%	79%	103%	96%
*Juniperus oblonga*	Root	88%	52%	73%	37%	106%	111%	104%	131%
	Leaf and fruit	92%	55%	57%	37%	45%	26%	100%	98%
	Stem	74%	86%	80%	72%	101%	104%	79%	99%
*Melandrium album*	Entire plant	83%	84%	68%	53%	80%	87%	88%	99%
*Mentha longifolia*	Entire plant	111%	87%	77%	67%	80%	50%	130%	90%
*Pimpinella peregrina*	Entire plant	95%	84%	75%	100%	94%	83%	107%	109%
*Polygonum hydropiper*	Entire plant	79%	82%	40%	37%	88%	89%	99%	95%
*Primula macrocalyx*	Entire plant	84%	114%	28%	37%	88%	114%	118%	106%
*Ruscus hyrcanus*	Leaf and fruit	89%	70%	90%	107%	101%	103%	82%	122%
*Scrophularia orientalis*	Entire plant	90%	83%	74%	39%	124%	76%	138%	94%
*Scutellaria orientalis*	Entire plant	121%	82%	65%	49%	48%	50%	45%	94%
*Solanum dulcamara*	Stem and root	86%	90%	80%	47%	89%	97%	71%	69%
	Entire plant	102%	90%	88%	80%	111%	84%	75%	115%
*Stachys lavandulifolia*	Entire plant	54%	76%	51%	39%	64%	71%	36%	76%
*Symphytum asperum*	Entire plant	44%	67%	44%	35%	60%	26%	82%	81%
*Thymus kotschyanus*	Entire plant	107%	72%	88%	75%	37%	24%	58%	68%
*Veratrum lobelianum*	Entire plant	104%	79%	86%	73%	100%	73%	92%	103%
*Zygophyllum fabago*	Entire plant	86%	73%	46%	53%	60%	60%	80%	86%

Three hexane extracts (*Achillea biebersteinii*, *Chaerophyllum bulbosum*, *Symphytum asperum*), 17 dichloromethane extracts (*Anchusa azurea*, *Camphorosma lessingii*, *Chaerophyllum bulbosum*, *Echium russicum*, *Elaeagnus orientalis*, *Glycyrrhiza glabra*, *Hypericum scabrum*, *Juncus effusus*, *Juniperus oblonga* (RT), *Juniperus oblonga* (LF + FR), *Melandrium album*, *Polygonum hydropiper*, *Primula macrocalyx*, *Solanum dulcamara* (ST + RT), *Stachys lavandulifolia*, *Symphytum asperum*, *Zygophyllum fabago*), 12 n-butanol extracts (*Anchusa azurea*, *Artocarpus altilis*, *Chaerophyllum bulbosum*, *Echium russicum*, *Glycyrrhiza glabra*, *Hypericum scabrum*, *Juniperus oblonga* (LF + FR), *Mentha longifolia*, *Scutellaria orientalis*, *Symphytum asperum*, *Thymus kotschyanus*, *Zygophyllum fabago*), and one aqueous extract (*Thymus kotschyanus*) showed inhibitory effects on both OAT1 and OAT3.

### IC_50_ values of plant extracts on OAT1- and OAT3-mediated 6-CF transport

**Figure 3:**
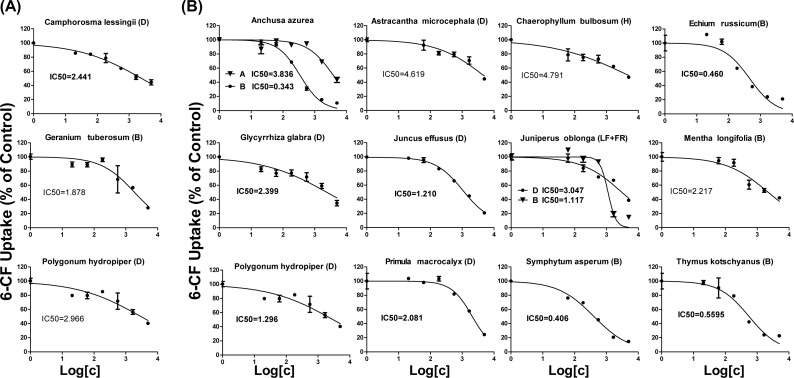
Dose-dependent inhibition of plant extracts on OAT1- and OAT3-mediated 6-CF uptake. Uptake of 6-CF (4 μM) in HEK-OAT1 and HEK-OAT3 cells were measured in the presence of increasing concentrations of plant extracts (0–5 μg∕ml). Each data points is the mean of triplicate values (*n* = 3) from a typical experiment and represents only the transporter-mediated transport. Means ± SD of triplicate determinations were given. The line represents a best fit of the data using nonlinear regression analysis. The unit for IC_50_ values was μg∕ml.

As shown in Table 1, 18 extracts for OAT1 and 25 extracts for OAT3 showed more than 50% inhibition. These 43 extracts were selected for further concentration-dependent inhibition studies. Some extracts did not show a good concentration-dependent inhibition on 6-CF uptake and thus were excluded from further investigation (Fig. S5). Three extracts (*Camphorosma lessingii* (D), *Geranium tuberosum* (B), *Polygonum hydropiper* (D)) were strong inhibitors for OAT1 with IC_50_ values being <5 μg∕ml (2.441, 2.966, and 1.878 μg∕ml, respectively) (Fig. 3A). Fourteen extracts (*Anchusa azurea* (A and B), *Astracantha microcephala* (D), *Chaerophyllum bulbosum* (H), *Echium russicum* (B), *Glycyrrhiza glabra* (D), *Juncus effusus* (D), *Juniperus oblonga* (LF + FR) (B and D), *Mentha longifolia* (B), *Polygonum hydropiper* (D), *Primula macrocalyx* (D), *Thymus kotschyanus* (B), *Symphytum asperum* (B)) were strong inhibitors of OAT3 with IC_50_ values being <5 μg∕ml (Fig. 3B).

### Interaction of *Juncus effusus* (D) with FS in rats

**Figure 4:**
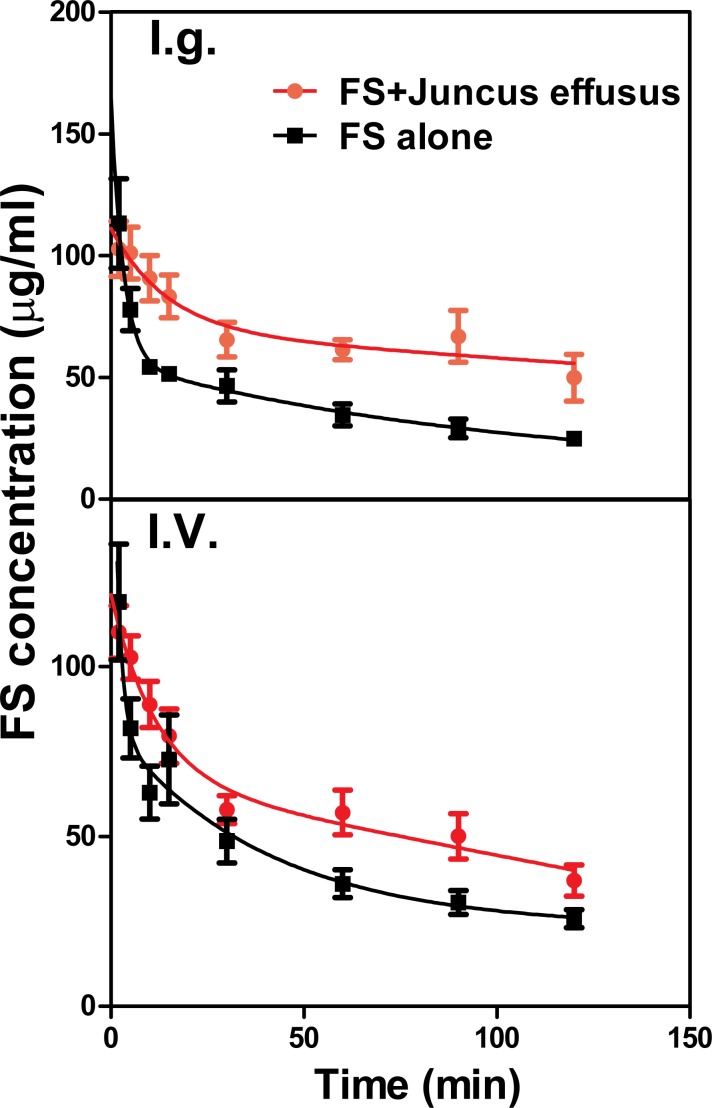
The plasma concentration–time course of FS in rats pretreated with *Juncus effusus* extract. Plasma concentration profile of furosemide in rats after tail vein administration of 10 mg/kg furosemide alone (black line) and furosemide with 5 min pre-treatment of 100 mg/kg *Juncus effusus* extract (red line) by oral gavage (A) and tail vein injection (B). Each data point represents the mean ± S.D. of 4–5 rats.

The mean plasma concentration–time profiles of FS in the presence and absence of *Juncus effusus* (D) in rats is shown in Fig. 4. Pharmacokinetic parameters of FS were summarized in Table 2. Results indicated that pretreatment with *Juncus effusus* (D) significantly altered the pharmacokinetics of FS in rats. The AUC_0–t_ values of FS were increased by 80% and 55% after oral and intravenous coadministration with *Juncus effusus* (D), respectively. Therefore, concomitant use of *Juncus effusus* (D) with substrates of OAT1 and OAT3 should require close monitoring for potential HDIs.

**Table 2:** Pharmacokinetic parameters of FS in rats following oral and intravenous administration of FS (10 mg/kg) in the presence and absence of Juncus effusus extract (100 mg/kg).

Parameters	FS	FS + Juncus effusus (oral)	FS + Juncus effusus (intravenous)
t_1∕2_(min)	107.3 ± 10.4	327.2 ± 18.1	101.5 ± 10.1
C_0_(μg∕ml)	181.713.5	105.8 ± 10.3	115.8 ± 10.8
AUC_0–t_ (μg∕ml ∗ min)	4588.967.7	8254.7 ± 90.9	7108.4 ± 84.3
AUC_0–inf_obs_(μg∕ml ∗ min)	10,000.8 ± 100.0	33,172.4 ± 182.1	12,465.3 ± 111.6
V _z_obs_ (L/kg ×10^−3^)	146.9 ± 12.1	123.6 ± 11.1	118.2 ± 10.9
Cl_obs_ (L/min/kg × 10^−3^)	1.0 ± 1.0	0.4 ± 0.6	0.8 ± 0.9

## Discussion

Plants are capable of synthesis and accumulation of a high diversity of secondary metabolites (SMs) or natural products (NPs) that are not essential for the actual metabolism or physiology of the plants (Wink, 2008). Many phenolic SMs, such as ellagic acid and caffeic acid, have been shown to be strong inhibitors of OATs (Uwai et al., 2011; Whitley, Sweet & Walle, 2005). Flavonoid SMs, such as morin and silybin, were also demonstrated to interact with OATs. Additionally, a recent study reported that several anthraquinones from rhubarb were strong inhibitors of human OATs (Ma et al., 2014). Although OATs are present in various tissues, the investigation of DDIs attributable to OAT inhibition has so far concentrated on the kidney. In particular, OAT1 and OAT3, which are highly expressed in the kidney, have been shown to be involved in several DDIs (Lepist & Ray, 2012) (Motohashi et al., 2002). So far, few studies have been conducted to elucidate the interactions of herbal extracts with OAT1 and OAT3.

In the present study, 172 extracts from 37 medicinal and economic plants were screened for the effect on OAT1- and OAT3-mediated transport. These plants grow in different parts of the world, and are frequently consumed as herbal medicine, fruits, vegetables, and drinks (Table 1). Among the 172 extracts screened, 59 plant extracts showed an inhibition to OAT1, and 55 plant extracts showed an inhibition to OAT3. OAT3 shares a 51% homology to OAT1 (Cha et al., 2001). Therefore, it is not surprisingly that 33 extracts were able to inhibit both OAT1 and OAT3.

Concentration-dependent inhibition of 6-CF uptake was further conducted for 45 plant extracts with more than 50% inhibition to OATs. Three extracts were identified as strong inhibitors of OAT1 with IC_50_ values being <5 μg∕ml. *Geranium tuberosum* (B) was the strongest inhibitor for OAT1, with IC_50_ value being 1.878 μg∕ml (Table 1). *Camphorosma lessingii* (D) and *Polygonum hydropiper* (D) also markedly inhibited OAT1. *Geranium tuberosum* has been used as folk medicine to treat fever, tonsillitis, cough, urticaria, dysentery, kidney pain, and gastrointestinal ailments (Calzada et al., 1999). *Camphorosma lessingii* is used in Central Asia externally to treat fungal skin diseases and internally to treat rheumatism (Takeuchi et al., 1977). *Polygonum hydropiper* has historically been used to treat several diseases, such as diarrhea, piles, bleeding, and parasitic worms, and colic pain (Xiao et al., 2016). Our data suggest that concomitant use of these three herbs may increase the risk of HDIs with OAT1 substrate drugs.

Fourteen extracts showed a strong inhibition to OAT3 with IC_50_ values being <5 μg∕ml. *Anchusa azurea* is widely distributed in the Mediterranean region, and has shown antitussive and anti-inflammatory properties (Kuruuzum-Uz et al., 2012). *Astracantha microcephala* and *Chaerophyllum bulbosum* are used in the food industry. *Echium russicum* is a rare plant growing in central and southeastern Europe, and has shown antioxidant and antimicrobial activities (Niciforovic et al., 2010). *Glycyrrhiza glabra* is the source of licorice which has been widely used in tobacco and food industry (Arora et al., 2016). *Juncus effusus* is a well-known traditional Chinese medicine used as a sedative, anxiolytic and antipyretic to treat fidgetiness and insomnia (Ma et al., 2015). *Juniperus oblonga* is a medicinal plant with diuretic and antiscorbutic effects (Pisarev et al., 2011). *Mentha longifolia* is widely used in traditional medicine to treat many gastrointestinal disorders such as abdominal pain, diarrhea, ulcers, and gut spasm (Murad, Abdallah & Ali, 2016). *Primula macrocalyx* has been used as a kind of spice due to its peppery taste. *Symphytum asperum* has demonstrated antioxidant and anti-inflammatory properties (Barthomeuf et al., 2001). *Thymus kotschyanus* has been traditionally implemented in the treatment of wounds, throat and gum infections and gastro-intestinal disorder (Rasooli & Mirmostafa, 2003). Our data suggest that potential HDIs might exist when consuming these herbs with OAT3 substrate drugs.

Although the *in vitro* data suggest a potential HDI of these extracts, the *in vivo* environment is more complex. Furosemide, a loop or high-ceiling diuretic, is eliminated mainly by renal excretion in which OAT1 and three play major roles (Brandoni et al., 2006). The *in vitro* study showed that *Juncus effusus* (D) was a strong inhibitor of OAT3 with IC_50_ value being 1.21 μg∕ml, and also slightly inhibited OAT1. In the preliminary study, we found that 100 mg/kg of *Juncus effusus* (D) did not cause any obvious toxic response in mice or rats. Both oral and intravenous co-administration of *Juncus effusus* (D) markedly increased the AUC_0–t_ of FS in rats. Therefore, consideration must be given to the potential clinical interaction of *Juncus effusus* (D) with drug substrates of OATs.

## Conclusion

In summary, we have investigated the inhibitory effects of 172 plant extracts from 37 medicinal plants on OAT1 and OAT3 *in vitro*, and identified four strong inhibitors for OAT1 and 14 strong inhibitors for OAT3. The *in vivo* study showed that *Juncus effusus* markedly altered the pharmacokinetics of FS. It is worthwhile to note that a number of the plants in the present study are used as foods or beverages rather than drugs, and people consuming them may not realize the potential HDIs. The future studies will focus on the isolation and characterization of the active ingredients responsible for the inhibitory effects of these extracts. Given the important physiological and pharmacological role of OATs, the information obtained in this study will not only help to prevent the potential HDIs, but also provide a mechanistic view about the therapeutic applications of the herbs.

##  Supplemental Information

10.7717/peerj.3333/supp-1 Supplemental Information 1 Supplementary figures and tables

10.7717/peerj.3333/supp-2 Supplemental Information 2 Raw data for Figures 1–4

10.7717/peerj.3333/supp-3 Supplemental Information 3 Raw data for Supplementary Figure 5

10.7717/peerj.3333/supp-4 Supplemental Information 4 Raw data for reproducibility test on those plant extracts showing strong OAT inhibition For the initial screening, those plant extracts showing more than 50% inhibition on OAT-mediated uptake were further repeated for the uptake experiment.

## Additional Information and Declarations

### Data Availability

The following information was supplied regarding data availability: The raw data has been supplied as Supplementary File.

## References

[ref-1] Arora P, Wani ZA, Nalli Y, Ali A, Riyaz-Ul-Hassan S (2016). Antimicrobial potential of thiodiketopiperazine derivatives produced by phoma sp., an endophyte of glycyrrhiza glabra linn. Microbial Ecology.

[ref-2] Barthomeuf CM, Debiton E, Barbakadze VV, Kemertelidze EP (2001). Evaluation of the dietetic and therapeutic potential of a high molecular weight hydroxycinnamate-derived polymer from Symphytum asperum Lepech. Regarding its antioxidant, antilipoperoxidant, antiinflammatory, and cytotoxic properties. Journal of Agricultural and Food Chemistry.

[ref-3] Bent S (2008). Herbal medicine in the United States: review of efficacy, safety, and regulation: grand rounds at University of California, San Francisco Medical Center. Journal of General Internal Medicine.

[ref-4] Bilgi N, Bell K, Ananthakrishnan AN, Atallah E (2010). Imatinib and Panax ginseng: a potential interaction resulting in liver toxicity. The Annals of Pharmacotherapy.

[ref-5] Brandoni A, Villar SR, Picena JC, Anzai N, Endou H, Torres AM (2006). Expression of rat renal cortical OAT1 and OAT3 in response to acute biliary obstruction. Hepatology.

[ref-6] Burckhardt G (2012). Drug transport by Organic Anion Transporters (OATs). Pharmacology and Therapeutics.

[ref-7] Bush TM, Rayburn KS, Holloway SW, Sanchez-Yamamoto DS, Allen BL, Lam T, So BK, Tran de H, Greyber ER, Kantor S, Roth LW (2007). Adverse interactions between herbal and dietary substances and prescription medications: a clinical survey. Alternative Therapies in Health and Medicine.

[ref-8] Calzada F, Cerda-Garcia-Rojas CM, Meckes M, Cedillo-Rivera R, Bye R, Mata R (1999). Geranins A and B, new antiprotozoal A-type proanthocyanidins from Geranium niveum. Journal of Natural Products.

[ref-9] Cha SH, Sekine T, Fukushima JI, Kanai Y, Kobayashi Y, Goya T, Endou H (2001). Identification and characterization of human organic anion transporter 3 expressing predominantly in the kidney. Molecular Pharmacology.

[ref-10] Chen Y, Li S, Brown C, Cheatham S, Castro RA, Leabman MK, Urban TJ, Chen L, Yee SW, Choi JH, Huang Y, Brett CM, Burchard EG, Giacomini KM (2009). Effect of genetic variation in the organic cation transporter 2 on the renal elimination of metformin. Pharmacogenet Genomics.

[ref-11] Duan P, Li S, Ai N, Hu L, Welsh WJ, You G (2012). Potent inhibitors of human organic anion transporters 1 and 3 from clinical drug libraries: discovery and molecular characterization. Molecular Pharmaceutics.

[ref-12] Eraly SA, Vallon V, Vaughn DA, Gangoiti JA, Richter K, Nagle M, Monte JC, Rieg T, Truong DM, Long JM, Barshop BA, Kaler G, Nigam SK (2006). Decreased renal organic anion secretion and plasma accumulation of endogenous organic anions in OAT1 knock-out mice. Journal of Biological Chemistry.

[ref-13] Hsueh CH, Yoshida K, Zhao P, Meyer TW, Zhang L, Huang SM, Giacomini KM (2016). Identification and quantitative assessment of uremic solutes as inhibitors of renal organic anion transporters, OAT1 and OAT3. Molecular Pharmaceutics.

[ref-14] Jankowski A, Skorek-Jankowska A, Lamparczyk H (1997). Determination and pharmacokinetics of a furosemide-amiloride drug combination. Journal of Chromatography B.

[ref-15] Kuruuzum-Uz A, Suleyman H, Cadirci E, Guvenalp Z, Demirezer LO (2012). Investigation on anti-inflammatory and antiulcer activities of Anchusa azurea extracts and their major constituent rosmarinic acid. Zeitschrift für Naturforschung C.

[ref-16] Lepist EI, Ray AS (2012). Renal drug-drug interactions: what we have learned and where we are going. Expert Opinion on Drug Metabolism & Toxicology.

[ref-17] Ma W, Liu F, Ding YY, Zhang Y, Li N (2015). Four new phenanthrenoid dimers from Juncus effusus L. with cytotoxic and anti-inflammatory activities. Fitoterapia.

[ref-18] Ma L, Zhao L, Hu H, Qin Y, Bian Y, Jiang H, Zhou H, Yu L, Zeng S (2014). Interaction of five anthraquinones from rhubarb with human organic anion transporter 1 (SLC22A6) and 3 (SLC22A8) and drug-drug interaction in rats. Journal of Ethnopharmacology.

[ref-19] Motohashi H, Sakurai Y, Saito H, Masuda S, Urakami Y, Goto M, Fukatsu A, Ogawa O, Inui K (2002). Gene expression levels and immunolocalization of organic ion transporters in the human kidney. Journal of the American Society of Nephrology.

[ref-20] Murad HA, Abdallah HM, Ali SS (2016). Mentha longifolia protects against acetic-acid induced colitis in rats. Journal of Ethnopharmacology.

[ref-21] Niciforovic N, Mihailovic V, Maskovic P, Solujic S, Stojkovic A, Muratspahic D (2010). Antioxidant activity of selected plant species; potential new sources of natural antioxidants. Food and Chemical Toxicology.

[ref-22] Nigam SK, Bush KT, Martovetsky G, Ahn SY, Liu HC, Richard E, Bhatnagar V, Wu W (2015). The organic anion transporter (OAT) family: a systems biology perspective. Physiological Reviews.

[ref-23] Perwitasari O, Yan X, Johnson S, White C, Brooks P, Tompkins SM, Tripp RA (2013). Targeting organic anion transporter 3 with probenecid as a novel anti-influenza a virus strategy. Antimicrobial Agents and Chemotherapy.

[ref-24] Pisarev DI, Novikov OO, Novikova MY, Zhilyakova ET (2011). Flavonoid composition of Juniperus oblonga Bieb. Bulletin of Experimental Biology and Medicine.

[ref-25] Rasooli I, Mirmostafa SA (2003). Bacterial susceptibility to and chemical composition of essential oils from Thymus kotschyanus and Thymus persicus. Journal of Agricultural and Food Chemistry.

[ref-26] Sweet DH, Miller DS, Pritchard JB, Fujiwara Y, Beier DR, Nigam SK (2002). Impaired organic anion transport in kidney and choroid plexus of organic anion transporter 3 (Oat3 (Slc22a8)) knockout mice. Journal of Biological Chemistry.

[ref-27] Takeuchi T, Nakagawa Y, Ogawa M, Kawachi T, Sugimura T (1977). Immunoreactivities of alpha-amylase of humans and rats. Clinica Chimica Acta.

[ref-28] Uwai Y, Ozeki Y, Isaka T, Honjo H, Iwamoto K (2011). Inhibitory effect of caffeic acid on human organic anion transporters hOAT1 and hOAT3: a novel candidate for food-drug interaction. Drug Metabolism and Pharmacokinetics.

[ref-29] Vallon V, Eraly SA, Wikoff WR, Rieg T, Kaler G, Truong DM, Ahn SY, Mahapatra NR, Mahata SK, Gangoiti JA, Wu W, Barshop BA, Siuzdak G, Nigam SK (2008). Organic anion transporter 3 contributes to the regulation of blood pressure. Journal of the American Society of Nephrology.

[ref-30] Wang L, Sweet DH (2012). Active hydrophilic components of the medicinal herb salvia miltiorrhiza (Danshen) potently inhibit organic anion transporters 1 (Slc22a6) and 3 (Slc22a8). Evidence-Based Complementary and Alternative Medicine.

[ref-31] Whitley AC, Sweet DH, Walle T (2005). The dietary polyphenol ellagic acid is a potent inhibitor of hOAT1. Drug Metabolism and Disposition: The Biological Fate of Chemicals.

[ref-32] Wink M (2008). Evolutionary advantage and molecular modes of action of multi-component mixtures used in phytomedicine. Current Drug Metabolism.

[ref-33] Xiao H, Rao Ravu R, Tekwani BL, Li W, Liu WB, Jacob MR, Khan SI, Cai X, Peng CY, Khan IA, Li XC, Wang W (2016). Biological evaluation of phytoconstituents from Polygonum hydropiper. Natural Product Research.

[ref-34] Zhang Y, Huo M, Zhou J, Xie S (2010). PKSolver: an add-in program for pharmacokinetic and pharmacodynamic data analysis in Microsoft Excel. Computer Methods and Programs in Biomedicine.

