# Supplementary material for: Interactions of 172 plant extracts with human organic anion transporter 1 (SLC22A6) and 3 (SLC22A8): a study on herb-drug interactions
Source: PeerJ. 2017 May 25;5:e3333. doi: 10.7717/peerj.3333 (PMC5446775; doi:10.7717/peerj.3333)
Supplement: Supplemental Information 1 [file peerj-05-3333-s001.doc]

**Supplementary Table 1. The 37 medicinal and economic plants investigated in the present study.**

| **Botanical name** | **Function** | **Growing area** |
| --- | --- | --- |
|
| *Achillea biebersteinii* | Abdominal pain, wounds and stomachache | Europe, Asia, and North America |
| *Ambrosia artemisiifolia* | A traditional medicinal plant for Native American tribes | Americas |
| *Anchusa azurea* | Health disorders, antitussive and anti-inflammatory | Mediterranean region |
| *Artocarpus altilis* | Liver cirrhosis, hypertension, and diabetes | Native to Indonesia |
| *Atraphaxis spinosa* | Eaten as a food or used in making sweetmeats | Egypt |
| *Astracantha microcephala* | Gum tragacanth - used as a thickener in confections | Iraq |
| *Buxus hyrcana* | Anti-plasmodial activity acetylcholinesterase-inhibitor immunosuppressive, antioxidant, anti-HIV, antifungal | Iran |
| *Caccinia macranthera* | Dermal infections, liver disorders, dyspepsia | Iranian |
| *Caltha polypetala* | Vegetable | Eastern Anatolia Region, Turkey |
| *Camphorosma lessingii* | Fungal skin diseases, diuretic, diaphoretic, rheumatism | Central and south Asia |
| *Cardaria boissieri* | Jellies made from the fruits safe to eat. Rheumatism and pulmonary illness | United States and Mexico |
| *Chaerophyllum bulbosum* | Vegetable | [Europe and Western Asia](https://en.wikipedia.org/wiki/Western_Asia) |
| *Crataegus orientalis* | Fruits as food | Mediterranean, Turkey, Crimea, and Iran |
| *Echium russicum* | Extracts demonstrated antioxidant and antimicrobial activities | Europe |
| *Elaeagnus orientalis* | Tumours, certain types of cancer, and viral diseases | North America, Europe, India, Pakistan |
| *Eremostachys macrophylla macrophylla* | Analgesic and anti-inflammatory agents | Iran |
| *Galium verum* | Diuretic, diaphoretic, spasmolytic and skin injury | Europe, North Africa, Turkey |
| *Geranium tuberosum* | Eaten in Erzurum | Mediterranean and  East to Iran |
| *Glycyrrhiza glabra* | Traditional kampo medicine to treat a variety of diseases | Southwest and Central Asia |
| *Hypericum androsaemum* | Diuretic, hepatoprotector, cholagogue, anti-kidney failure properties | Europe, North Africa and Asia Minor |
| *Hypericum scabrum* | Bladder, intestinal, and heart diseases, rheumatism, and cystitis | Turkey |
| *Juncus effusus* | Antiphlogistic, antipyretic, antipyretic, sedative, and diuretic | Europe, Asia, Africa, America |
| *Juniperus oblonga* | Diuretic and antiscorbutic | Iran, Turkey, Caucasus |
| *Melandrium album* | An infusion as a physic | Europe, Western Asia and Northern Africa |
| *Mentha longifolia* | Fevers, headaches, digestive disorders and various minor ailments | Turkish flora |
| *Pimpinella peregrina* | Cooked with oil, vegetable pie | Albertkanaal |
| *Polygonum hydropiper* | Spice | Southeast Asia |
| *Primula macrocalyx* | Paralysis tumor, wound healing, and blood diseases | Russian Federation |
| *Ruscus hyrcanus* | Diuretic, appetizer, antilaxative, vasoconstrictor, antibleeding, antinephritis | Azerbaijan |
| *Scrophularia orientalis* | The extract reduced cell survival and increased calcium levels in NB cells |  |
| *Scutellaria orientalis* | Antidiarrheaic, haemostatic, tonic and for healing | Western Turkey |
| *Solanum dulcamara* | Cancers and warts | Europe and Asia |
| *Stachys lavandulifolia* | Anxiolytic and sedative | Iran |
| *Symphytum asperum* | Disorders, mainly fractures and wounds | Asia |
| *Thymus kotschyanus* | Wounds, throat and gum infections and gastro-intestinal disorder | Iranian |
| *Veratrum lobelianum* | Folk medicine (Chinese medicine “Li-lu”) | Temperate northern hemisphere |
| *Zygophyllum fabago* | Antitussive, expectorant, anti-inflammatory and removing pains | Asia and Europe |

**Supplementary Table 2. RT-PCR primer sequences.** Primers were designed with Primer3 software (version 4). Total RNA was isolated with RNAiso Plus reagent (Takara Biotechnology, Dalian, China) according to the manufacturer’s protocol. RNA was then reverse transcribed to cDNA using a SuperScript II RT kit (Invitrogen). Quantitative reverse transcriptase PCR analysis was carried out using SYBR green PCR mastermix (ABI Inc.). Values were normalized to glyceraldehyde-3-phosphate dehydrogenase (GADPH).

| **Gene** | **Primer(5'-3')** | |
| --- | --- | --- |
|  | **F** | **R** |
| OAT1 | CCAAGGCTGTGGGGAGAAGGC | AGAGGAAGAGGTGGCGGAGGG |
| OAT3 | GGGGTCTGGGTCTGGCTCAAC | GTGCTCTCCCAGCCAGTCTCC |
| GAPDH | GAAGGTCGGAGTCAACGGATT | CGCTCCTGGAAGATGGTGAT |

**Supplementary Figure 1. The procedure to prepare plant extracts.** In brief, fresh plant samples were dried in air and milled to a coarse powder. A portion of each sample (1 kg dry weight) was extracted with methanol (3x4 L). The solvent was removed *in vacuo* to obtain viscous oil, which was then dispersed in 1L of methanol: water (9:1) and extracted with n-hexane (3x1L) to obtain the hexane (H) fraction. The methanol was removed from the hydro-alcoholic phase, which was then dispersed in distilled water (1L). This mixture was extracted sequentially with dichloromethane (3x1L) to get the dichloromethane (D) fraction, and with water saturated n-butanol (3x1L) to obtain n-butanol (B) and aqueous (A) fractions, respectively. The final four fractions (H, D, B, and A) were individually evaporated to dryness *in vacuum*, and were maintained at -20˚C until use.

**Supplementary Figure 2. The mRNA expression of OAT1 in HEK-OAT1 cells and OAT3 in HEK-OAT3 cells.**

**Supplementary Figure 3. The reproducibility of uptake experiments in the present study.** To evaluate the reproducibility of cell uptake assays used in the present study, uptake of 6-CF (4 μM) in HEK-OAT1 and HEK-OAT3 cells were measured in the presence and absence of probenecid (100 μM) in three cell culture plates with four replicated wells per uptake experiment in each plate. Values were expressed as the percent of control. Means ± SD of four repeated determinations were given.

**Supplementary Figure 4. The effect of DMSO on OAT1- and OAT3-mediated 6-CF uptake.** (A) Uptake of 6-CF (4 μM) in HEK-OAT1 and HEK-OAT3 cells were measured in the presence of DMSO at various concentrations (0-2%), respectively. Values were given as percent of control. Means ± SD of triplicate determinations (n=3) were given. (B) A recent uptake experiment confirmed the effect of DMSO on 6-CF (4 μM) uptake in HEK-OAT3 cells. Values were given as the fluorescent unit. Means ± SD of triplicate determinations (n=3) were given.

**
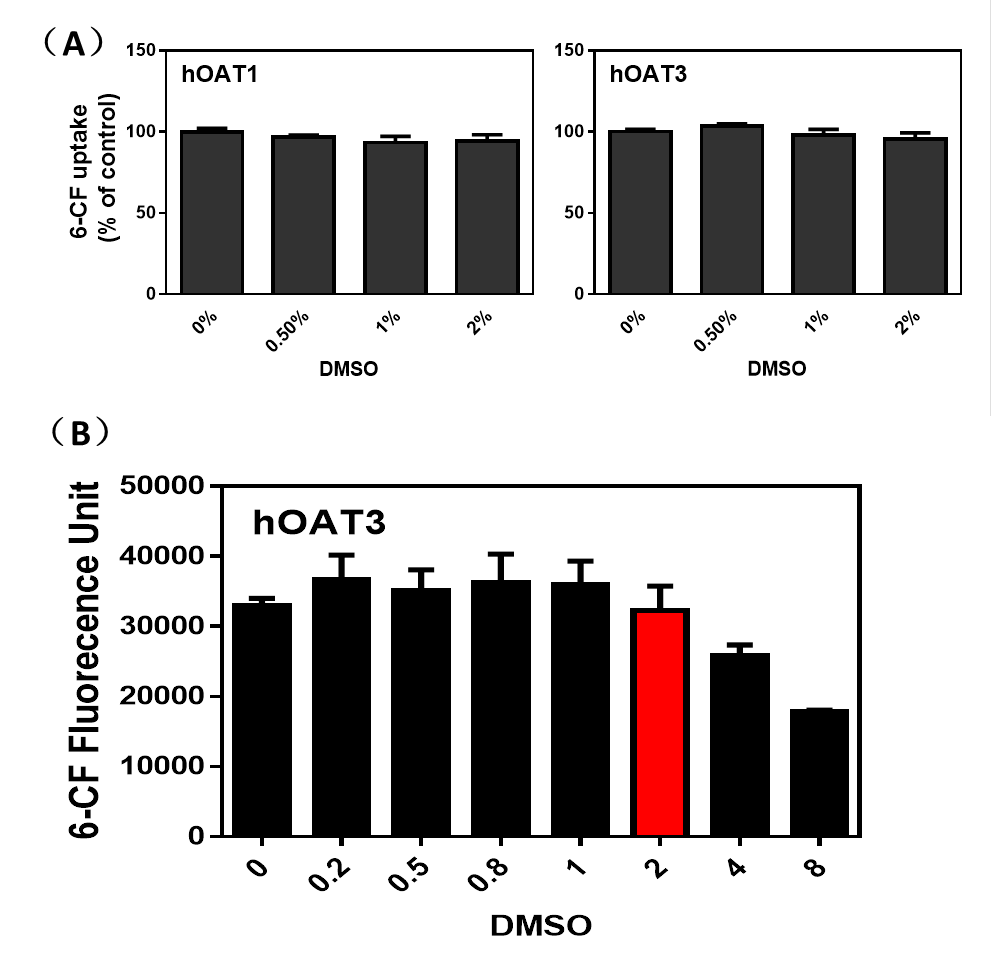
**

**Supplementary Figure 5. Dose-dependent inhibition of plant extracts on OAT1- and OAT3-mediated 6-CF uptake.** Uptake of 6-CF (4 μM) in HEK-OAT1 and HEK-OAT3 cells were measured in the presence of increasing concentrations of plant extracts (0.02-5 μg/ml). The unit of x-axis is the log value of the concentrations. Each data point is the mean of triplicate values (n=3) from a typical experiment and represents only the transporter-mediated transport by subtracting the values of control cells. The line represents a best fit of the data using nonlinear regression analysis. Means ± SD of triplicate determinations were given.

1. **OAT1**
2. **OAT3**
