# Supplementary material for: Interactions of 172 plant extracts with human organic anion transporter 1 (SLC22A6) and 3 (SLC22A8): a study on herb-drug interactions
Source: PeerJ. 2017 May 25;5:e3333. doi: 10.7717/peerj.3333 (PMC5446775; doi:10.7717/peerj.3333)
Supplement: Supplemental Information 2 [file peerj-05-3333-s002.docx]

**Figure 1**

**Figure 1A raw data**

Time course of 6-CF uptake in HEK-EV and HEK-OAT1 cells

| time (min) | HEK-EV | | | HEK-OAT1 | | |
| --- | --- | --- | --- | --- | --- | --- |
| 0 | 1 | 2 | 3 | 1 | 2 | 3 |
| 1 | 2.028983 | 2.127006 | 1.448797 | 103.0408 | 111.2811 | 116.9642 |
| 2 | 3.516795 | NA | 2.495629 | 184.2101 | 188.6327 | 203.3025 |
| 4 | 3.274365 | 1.898699 | NA | 263.4748 | 273.8167 | 274.8526 |
| 6 | 2.900359 | 5.178694 | 2.231402 | 366.3584 | 347.2089 | 351.4604 |
| 8 | 2.807204 | 2.226492 | 2.133867 | 433.3665 | 398.0952 | 430.7222 |
| 10 | 6.849454 | NA | 3.392078 | 468.8556 | 433.6342 | 503.1246 |
| 15 | 2.155927 | 2.367465 | 2.880028 | 515.439 | NA | 498.8523 |

Saturation curve of 6-CF uptake in HEK-OAT1 cells

| Conc (uM) | 1 | 2 | 3 |
| --- | --- | --- | --- |
| 500 | 180.965 | 184.434 | 169.8854 |
| 250 | 164.0673 | 159.2171 | 152.7382 |
| 125 | 148.3736 | 150.9828 | 144.0527 |
| 62.5 | 137.2491 | 143.8246 | 138.2214 |
| 31.25 | 121.9009 | 125.5377 | 115.7964 |
| 15.625 | 99.74324 | 104.681 | 93.41476 |
| 7.8125 | 70.10327 | 79.18506 | 68.4603 |
| 3.90625 | 46.16213 | 53.71017 | 47.46804 |
| 1.953125 | 27.86526 | 28.3406 | 28.05018 |
| 0.9765625 | 12.8274 | 13.23398 | 13.00434 |
| 0.4882813 | 7.173068 | 7.445915 | 6.987242 |
| 0 | 0 | 0 | 0 |

Time course of 6-CF uptake in HEK-EV and HEK-OAT3 cells

| Time (min) | EV | | | OAT3 | | |
| --- | --- | --- | --- | --- | --- | --- |
| 0 | -0.2959743 | -0.1638222 | -0.3041136 | -0.2934467 | -0.313935 | -0.3556867 |
| 1 | 0.1863895 | 0.1214707 | 0.1925655 | 15.96047 | 14.62697 | 12.88563 |
| 3 | 1.062053 | 1.737515 | 0.66243 | 23.66435 | 23.83295 | 22.4238 |
| 5 | 1.599855 | 1.945673 | 1.900472 | 30.90043 | 31.15759 | 28.04544 |
| 7 | 3.080659 | 4.379843 | 4.256661 | 39.99162 | 36.18999 | 41.91379 |
| 10 | 12.28623 | 7.712022 | 11.67251 | 50.23714 | 52.458 | 60.541 |
| 15 | 22.75172 | 12.80822 | 21.0992 | 52.72028 | 56.05032 | 66.23049 |
| 20 | 40.59988 | 70.13045 | 42.87365 | 77.99735 | 76.08685 | 111.922 |

Saturation curve of 6-CF uptake in HEK-OAT3 cells

| Conc (uM) | 1 | 2 | 3 |
| --- | --- | --- | --- |
| 500 | 59.66524 | 68.35542 | 60.31531 |
| 250 | 38.59276 | 48.68475 | 49.92839 |
| 125 | 31.12392 | 34.21449 | 34.21501 |
| 62.5 | 24.87196 | 28.10585 | 27.04858 |
| 31.25 | 16.86702 | 19.83209 | 17.41912 |
| 15.625 | 11.68126 | 13.79611 | 13.42072 |
| 7.8125 | 6.975257 | 7.312179 | 7.453115 |
| 3.90625 | 4.350202 | 4.47157 | 4.585364 |
| 1.953125 | 1.795322 | 1.575202 | 1.457688 |
| 0.9765625 | 0.6980292 | 0.5036659 | 0.6877155 |
| 0.4882813 | 0.3917566 | 0.3328885 | 0.4387769 |
| 0 | 0 | 0 | 0 |

**Figure 1B raw data**

Dose-dependent inhibition of probenecid on 6-CF uptake in HEK-OAT1 cells

| Conc (uM) | 1 | 2 | 3 |
| --- | --- | --- | --- |
| 0 | 103 | 99 | 98 |
| 1 | 100 | 95 | 95 |
| 5 | 89 | 85 | 88 |
| 10 | 78 | 70 | 86 |
| 50 | 37 | 37 | 39 |
| 75 | 25 | 23 | 25 |
| 100 | 19 | 17 | 20 |
| 200 | 8 | 7 | 7 |

Dose-dependent inhibition of probenecid on 6-CF uptake in HEK-OAT3 cells

| Conc (uM) | 1 | 2 | 3 |
| --- | --- | --- | --- |
| 0 | 99 | 99 | 102 |
| 1 | 93 | 89 | 90 |
| 5 | 65 | 64 | 62 |
| 10 | 46 | 44 | 44 |
| 50 | 17 | 16 | 12 |
| 75 | 13 | 8 | 13 |
| 100 | 9 | 11 | 10 |
| 200 | 6 | 5 | 6 |

**Figure 2 Raw Data**

Inhibitory effects of hexane extracts on OAT-mediated 6-CF.

| Plant extracts | OAT1 | | | OAT3 | | |
| --- | --- | --- | --- | --- | --- | --- |
| Zygophyllum fabago | 113.7676 | 37.31233 | 108.1423 | 78.17689 | 72.76936 | 67.8873 |
| Veratrum lobelianum | 102.9177 | 97.22022 | 110.561 | 75.80183 | 84.89329 | 77.3519 |
| Thymus kotschyanus | 108.9144 | 106.7108 | 104.6826 | 87.477 | 44.41782 | 83.57133 |
| Symphytum asperum | 37.79681 | 32.7139 | 60.73064 | 71.27805 | 74.34994 | 55.40131 |
| Stachys lavandulifolia | 43.31734 | 95.60529 | 22.91483 | 89.24559 | 58.64338 | 79.42172 |
| Solanum dulcamara(FL+FR+LF) | 99.82683 | 103.5925 | 103.9043 | 78.63404 | 97.36359 | 92.94591 |
| Solanum dulcamara(ST+RT) | 101.5605 | 49.31287 | 106.0375 | 93.8131 | 98.44233 | 78.52845 |
| Scutellaria orientalis | 96.50167 |  | 70.69672 | 80.28638 | 80.1764 | 85.95593 |
| Scrophularia orientalis | 86.78188 | 83.9394 | 98.09074 | 83.28025 | 83.40402 | 81.44392 |
| Ruscus hyrcanus | 82.3762 | 92.51748 | 92.36542 | 72.67318 | 66.80059 | 70.83691 |
| Primula macrocalyx | 76.80809 | 90.2246 | 85.4166 | 125.2015 | 108.0218 | 109.6833 |
| Polygonum hydropiper | 81.53288 | 82.2283 | 72.65329 | 95.26712 | 106.1628 | 45.62856 |
| Pimpinella peregrina | 88.69798 | 96.03211 | 101.2869 | 75.66944 | 83.54577 | 92.05032 |
| Mentha longifolia | 103.9104 | 114.3536 | 113.8688 | 57.9704 | 108.3781 | 93.69233 |
| Melandrium album | 83.42364 | 75.54787 | 90.30261 | 100.1055 | 47.70143 | 105.6294 |
| Juniperus oblonga(ST) | 99.80658 | 64.4488 | 57.38767 | 73.67492 | 88.35102 | 95.42716 |
| Juniperus oblonga(LF+FR) | 101.5735 | 107.6012 | 67.53038 | 77.85963 | 19.04816 | 68.13299 |
| Juniperus oblonga(RT) | 94.58553 | 80.33751 | 88.28139 | 52.29331 | 58.36474 | 45.41288 |
| Juncus effusus | 97.72713 | 104.2449 | 101.3444 | 81.96756 | 103.6836 | 91.44779 |
| Hypericum scabrum | 74.37407 | 40.28035 | 68.91111 | 101.2981 | 96.51504 | 91.15129 |
| Hypericum androsaemum | 88.56132 | 96.92023 | 106.223 | 91.09697 | 100.553 | 83.06477 |
| Glycyrrhiza glabra | 67.16585 | 71.35352 | 50.73243 | 73.43442 | 54.2588 | 100.5898 |
| Geranium tuberosum | 119.2241 | 118.5573 | 122.8431 | 114.8995 | 94.18563 | 125.6201 |
| Galium verum | 97.39282 | 103.1077 | 103.5359 | 96.31954 | 102.5215 | 102.9348 |
| Eremostachys macrophylla | 69.95899 | 79.16564 | 41.15397 | 58.43565 | 87.93982 | 107.1163 |
| Elaeagnus orientalis | 75.55225 | 85.92501 | 91.32572 | 76.35388 | 97.40312 | 106.1583 |
| Echium russicum | 117.5193 | 117.4056 | 117.0255 | 70.28778 | 81.71351 | 75.49758 |
| Crataegus orientalis | 106.1716 | 112.3637 | 111.8501 | 72.61932 | 94.20779 | 116.0332 |
| Chaerophyllum bulbosum | 76.1741 | 75.04522 | 79.97072 | 43.47613 | 36.84075 | 45.06956 |
| Cardaria boissieri | 87.17353 | 91.61787 | 88.04028 | 124.7271 | 111.8109 | 133.585 |
| Camphorosma lessingii | 71.19151 | 48.65791 | 82.33133 | 51.30245 | 90.51517 | 82.98932 |
| Caltha polypetala | 119.9142 | 87.52456 | 90.27864 | 95.66382 | 89.94669 | 85.04856 |
| Caccinia macranthera | 82.72212 | 93.08417 | 104.3725 | 123.8568 | 55.68137 | 126.9146 |
| Buxus hyrcana(ST) | 89.45786 | 87.89176 | 106.8327 | 64.7046 | 71.39233 | 66.41816 |
| Buxus hyrcana(RT) | 90.51127 | 85.45719 | 110.2522 | 82.77243 | 80.73389 | 69.61423 |
| Astracantha microcephala | 82.16692 | 106.0892 | 102.5066 | 73.57306 | 84.59324 | 82.8941 |
| Atraphaxis spinosa | 88.2778 | 95.70913 | 91.48955 | 95.41405 | 93.75538 | 101.9335 |
| Artocarpus altilis | 114.688 | 102.2952 | 90.88615 | 107.9261 | 99.57395 | 99.33897 |
| Anchusa azurea | 67.18588 | 57.6309 | 65.2505 | 92.34083 | 110.3963 | 88.56542 |
| Ambrosia artemisiifolia(ST) | 41.06857 | 60.28311 | 67.6913 | 83.64208 | 85.57677 | 53.26728 |
| Ambrosia artemisiifolia(RT) | 122.7238 | 92.10218 | 69.25107 | 105.891 | 71.73049 | 100.9648 |
| Ambrosia artemisiifolia(LF+FL) | 114.9221 | 106.7122 | 106.1051 | 117.0993 | 80.56998 | 100.6166 |
| Achillea biebersteinii | 48.62849 | 56.48881 | 44.59259 | 73.61225 | 73.42175 | 83.39035 |
| Prob | 6.532314 | 5.043622 | 4.34122 | 2.375008 | 2.144478 | 0.9311066 |
| Control | 103.3117 | 105.6123 | 91.07597 | 109.5225 | 84.86637 | 105.6111 |

Inhibitory effects of dichloromethane extracts on OAT-mediated 6-CF.

| Plant extracts | OAT1 | | | OAT3 | | |
| --- | --- | --- | --- | --- | --- | --- |
| Zygophyllum fabago | 27.59804 | 56.22597 | 53.42028 | 53.78635 | 52.82562 | 52.25877 |
| Veratrum lobelianum | 80.06327 | 89.47048 | 87.45394 | 82.87804 | 68.93992 | 66.31326 |
| Thymus kotschyanus | 85.5523 | 91.9343 | 87.49824 | 76.10503 | 80.68669 | 67.29603 |
| Symphytum asperum | 43.33827 | 65.5618 | 23.34816 | 37.7252 | 36.19672 | 31.02844 |
| Stachys lavandulifolia | 19.73124 | 66.84839 | 65.40024 | 55.76709 | 31.65549 | 30.66534 |
| Solanum dulcamara(FL+FR+LF) | 99.15301 | 68.43392 | 94.92003 | 82.95373 | 78.10593 | 79.08429 |
| Solanum dulcamara(ST+RT) | 77.3932 | 79.60358 | 83.63297 | 49.51991 | 46.70898 | 46.16307 |
| Scutellaria orientalis | 56.64825 | 93.51479 | 44.42402 | 50.87882 | 48.17945 | 49.18903 |
| Scrophularia orientalis | 91.71024 | 38.61791 | 90.65841 | 19.77786 | 48.59483 | 49.20314 |
| Ruscus hyrcanus | 86.53991 | 93.61438 | 90.79223 | 110.0824 | 105.3441 | 104.2941 |
| Primula macrocalyx | 30.14986 | 27.18677 | 25.56289 | 32.93535 | 34.43833 | 42.43123 |
| Polygonum hydropiper | 43.23547 | 39.05478 | 38.81147 | 41.05847 | 31.45664 | 38.71971 |
| Pimpinella peregrina | 69.8321 | 83.95564 | 72.26882 | 86.89054 | 107.2798 | 104.9174 |
| Mentha longifolia | 81.53725 | 60.18204 | 88.29435 | 68.48888 | 67.98115 | 65.34032 |
| Melandrium album | 61.20347 | 70.5779 | 71.80082 | 50.9089 | 52.49513 | 55.07927 |
| Juniperus oblonga(ST) | 96.52011 | 32.01616 | 111.4473 | 67.0592 | 71.30429 | 78.23625 |
| Juniperus oblonga(LF+FR) | 43.97299 | 74.7262 | 52.85833 | 35.76558 | 29.82591 | 45.28684 |
| Juniperus oblonga(RT) | 65.47197 | 71.65082 | 80.87327 | 49.32689 | 31.55541 | 30.51137 |
| Juncus effusus | 51.84093 | 57.02827 | 57.01082 | 20.35996 | 19.39948 | 24.46107 |
| Hypericum scabrum | 70.05379 | 76.82582 | 70.75533 | 60.98937 | 72.39793 | 81.74548 |
| Hypericum androsaemum | 66.3326 | 72.20074 | 64.73392 | 84.59441 | 77.22337 | 78.01701 |
| Glycyrrhiza glabra | 33.13912 | 45.63513 | 33.84665 | 22.10087 | 37.34037 | 20.94494 |
| Geranium tuberosum | 84.20066 | 84.80819 | 43.8485 | 102.0469 | 66.47106 | 67.67766 |
| Galium verum | 106.073 | 80.24926 | 82.36829 | 80.91951 | 73.45114 | 64.55804 |
| Eremostachys macrophylla | 69.36298 | 63.77317 | 73.28807 | 83.49269 | 72.43634 | 74.67867 |
| Elaeagnus orientalis | 64.81615 | 74.42634 | 78.15232 | 67.55584 | 63.30811 | 77.63354 |
| Echium russicum | 66.80659 | 70.10341 | 43.44865 | 52.87803 | 54.19385 | 52.94388 |
| Crataegus orientalis | 105.2999 | 91.26198 | 110.6605 | 128.1867 | 115.8235 | 79.36168 |
| Chaerophyllum bulbosum | 75.53594 | 78.61839 | 85.11264 | 61.3906 | 64.79115 | 69.37577 |
| Cardaria boissieri | 84.88892 | 88.7657 | 89.40211 | 86.59635 | 85.56895 | 46.86088 |
| Camphorosma lessingii | 35.00241 | 36.87897 | 39.85289 | 32.88736 | 56.19777 | 44.83707 |
| Caltha polypetala | 94.03387 | 89.88293 | 103.3213 | 43.02135 | 36.65294 | 57.49342 |
| Caccinia macranthera | 93.33366 | 110.7408 | 112.806 | 67.58233 | 65.48995 | 64.01643 |
| Buxus hyrcana(ST) | 76.86957 | 87.62789 | 86.30496 | 79.44674 | 84.41594 | 78.97823 |
| Buxus hyrcana(RT) | 91.31066 | 86.69095 | 89.50469 | 87.55126 | 83.49867 | 77.16439 |
| Astracantha microcephala | 83.13564 | 102.29719 | 93.69594 | 26.33608 | 52.69063 | 59.1931 |
| Atraphaxis spinosa | 38.56449 | 49.98581 | 49.9301 | 99.2029 | 68.43759 | 31.6458 |
| Artocarpus altilis | 71.61031 | 74.84531 | 86.90019 | 95.72966 | 90.50872 | 85.43311 |
| Anchusa azurea | 85.17166 | 66.75956 | 77.23537 | 49.60519 | 47.22381 | 48.79687 |
| Ambrosia artemisiifolia(ST) | 87.29469 | 31.65115 | 55.43914 | 55.8837 | 54.31203 | 51.8069 |
| Ambrosia artemisiifolia(RT) | 62.47402 | 26.81816 | 109.5984 | 77.46736 | 82.88041 | 38.58452 |
| Ambrosia artemisiifolia(LF+FL) | 90.8924 | 72.11136 | 100.6902 | 73.49079 | 40.2905 | 76.42949 |
| Achillea biebersteinii | 62.13155 | 69.61619 | 43.88175 | 66.8102 | 78.94389 | 39.62218 |
| Prob | 6.532314 | 5.043622 | 4.34122 | 2.375008 | 2.144478 | 0.9311066 |
| Control | 103.3117 | 105.6123 | 91.07597 | 109.5225 | 84.86637 | 105.6111 |

Inhibitory effects of butanol extracts on OAT-mediated 6-CF.

| Plant extracts | OAT1 | | | OAT3 | | |
| --- | --- | --- | --- | --- | --- | --- |
| Zygophyllum fabago | 55.35205 | 67.03495 | 58.71371 | 55.35205 | 67.03495 | 58.71371 |
| Veratrum lobelianum | 90.71254 | 101.2228 | 108.5126 | 53.76029 | 84.52116 | 81.28927 |
| Thymus kotschyanus | 37.34972 | 42.44303 | 31.40811 | 23.65334 | 25.12997 | 23.03815 |
| Symphytum asperum | 40.9749 | 71.63514 | 68.40097 | 20.67449 | 26.74038 | 30.0177 |
| Stachys lavandulifolia | 57.96638 | 33.97192 | 98.76776 | 93.89387 | 31.10998 | 87.6358 |
| Solanum dulcamara(FL+FR+LF) | 98.26534 | 112.1531 | 121.1721 | 84.56095 | 91.71662 | 76.88375 |
| Solanum dulcamara(ST+RT) | 56.26115 | 104.8892 | 105.6353 | 91.47804 | 98.58589 | 100.1383 |
| Scutellaria orientalis | 18.1494 | 59.95569 | 65.44749 | 56.80607 | 55.77062 | 37.5606 |
| Scrophularia orientalis | 125.1286 | 134.2814 | 112.6073 | 81.55656 | 69.65805 | 77.81239 |
| Ruscus hyrcanus | 101.3752 | 102.6813 | 99.98959 | 106.2999 | 101.2211 | 100.9731 |
| Primula macrocalyx | 96.1005 | 77.89208 | 88.65947 | 114.4949 | 110.3519 | 116.7398 |
| Polygonum hydropiper | 83.86374 | 87.43736 | 93.62826 | 84.07639 | 91.58201 | 92.07062 |
| Pimpinella peregrina | 93.99663 | 93.23954 | 95.52177 | 66.97712 | 90.58837 | 90.84365 |
| Mentha longifolia | 89.00917 | 72.84582 | 77.67917 | 56.13655 | 37.63287 | 57.37182 |
| Melandrium album | 75.51553 | 82.91181 | 80.68883 | 80.92462 | 98.88441 | 81.32797 |
| Juniperus oblonga(ST) | 117.3105 | 111.2586 | 75.67825 | 98.80106 | 118.8104 | 95.02572 |
| Juniperus oblonga(LF+FR) | 43.15617 | 28.06646 | 63.42772 | 20.28378 | 24.4887 | 32.07473 |
| Juniperus oblonga(RT) | 105.0331 | 103.5626 | 109.8011 | 108.5704 | 117.1121 | 106.9933 |
| Juncus effusus | 82.00275 | 99.78634 | 105.0889 | 75.7953 | 78.17053 | 82.3593 |
| Hypericum scabrum | 83.21949 | 75.5877 | 85.44129 | 67.06617 | 70.82459 | 55.7146 |
| Hypericum androsaemum | 70.46868 | 82.70829 | 67.77299 | 105.1661 | 123.9051 | 105.3748 |
| Glycyrrhiza glabra | 29.16114 | 70.87321 | 66.34656 | 62.70164 | 32.22073 | 63.82489 |
| Geranium tuberosum | 26.24687 | 31.75316 | 31.21504 | 85.00226 | 81.47581 | 77.06885 |
| Galium verum | 112.0311 | 122.5155 | 92.82077 | 62.83581 | 93.65983 | 89.121 |
| Eremostachys macrophylla | 74.15914 | 86.37247 | 81.80477 | 104.873 | 57.3684 | 105.7922 |
| Elaeagnus orientalis | 70.24389 | 30.11412 | 88.85131 | 83.03449 | 61.29753 | 104.3529 |
| Echium russicum | 49.43271 | 47.17757 | 49.1091 | 14.6907 | 18.36473 | 18.17486 |
| Crataegus orientalis | 115.78 | 117.188 | 123.5844 | 136.7693 | 151.1918 | 139.9973 |
| Chaerophyllum bulbosum | 60.03444 | 63.63833 | 69.67157 | 67.30427 | 73.26978 | 60.10852 |
| Cardaria boissieri | 88.7697 | 92.88077 | 80.11485 | 109.1806 | 63.04104 | 71.09637 |
| Camphorosma lessingii | 74.72795 | 72.70246 | 74.45948 | 104.9484 | 100.4232 | 110.3563 |
| Caltha polypetala | 94.37042 | 104.3023 | 110.6532 | 53.4335 | 89.74453 | 88.15641 |
| Caccinia macranthera | 95.44688 | 106.0687 | 115.0985 | 60.01449 | 62.45713 | 62.75998 |
| Buxus hyrcana(ST) | 107.8115 | 107.1385 | 105.5439 | 123.4715 | 120.2572 | 132.2106 |
| Buxus hyrcana(RT) | 86.00412 | 83.90161 | 82.53861 | 113.2195 | 119.5446 | 117.3602 |
| Astracantha microcephala | 69.67049 | 106.8766 | 112.9702 | 80.26524 | 72.07619 | 75.10836 |
| Atraphaxis spinosa | 103.1079 | 118.742 | 116.94 | 124.2859 | 126.973 | 124.5563 |
| Artocarpus altilis | 46.87549 | 50.46547 | 50.78616 | 53.32794 | 45.86805 | 57.23556 |
| Anchusa azurea | 40.12601 | 54.96886 | 78.64923 | 17.29192 | 19.00482 | 8.565373 |
| Ambrosia artemisiifolia(ST) | 86.56859 | 65.64904 | 22.37221 | 81.13244 | 35.80917 | 67.3845 |
| Ambrosia artemisiifolia(RT) | 90.10248 | 122.7737 | 36.83625 | 88.66453 | 91.7184 | 91.2535 |
| Ambrosia artemisiifolia(LF+FL) | 79.91917 | 101.2225 | 106.395 | 97.17534 | 74.15236 | 95.36104 |
| Achillea biebersteinii | 91.52195 | 78.64032 | 79.72174 | 81.0513 | 79.46095 | 117.6333 |
| Prob | 6.532314 | 5.043622 | 4.34122 | 2.375008 | 2.144478 | 0.9311066 |
| Control | 103.3117 | 105.6123 | 91.07597 | 109.5225 | 84.86637 | 105.6111 |

Inhibitory effects of aqueous extracts on OAT-mediated 6-CF.

| Plant extracts | OAT1 | | | OAT3 | | |
| --- | --- | --- | --- | --- | --- | --- |
| Zygophyllum fabago | 103.5709 | 47.99552 | 88.42381 | 95.9849 | 75.99552 | 86.24499 |
| Veratrum lobelianum | 64.13705 | 110.76 | 101.3134 | 126.2009 | 76.34211 | 105.8856 |
| Thymus kotschyanus | 55.96744 | 65.38826 | 52.77115 | 68.66603 | 77.75542 | 56.79598 |
| Symphytum asperum | 35.71341 | 104.5613 | 106.4774 | 74.17856 | 85.34276 | 82.41585 |
| Stachys lavandulifolia | 62.18596 | 32.36291 | 12.73491 | 84.31423 | 83.15965 | 60.02294 |
| Solanum dulcamara(FL+FR+LF) | 79.85893 | 75.1522 | 70.35584 | 123.1793 | 106.6661 | 113.982 |
| Solanum dulcamara(ST+RT) | 67.46167 | 68.38747 | 77.90107 | 48.59644 | 68.46324 | 91.38204 |
| Scutellaria orientalis | 74.39145 | 29.89373 | 29.74027 | 99.30212 | 93.27077 | 90.54337 |
| Scrophularia orientalis | 134.2542 | 141.0731 | 139.1868 | 94.36215 | 96.75312 | 89.87765 |
| Ruscus hyrcanus | 98.23514 | 77.42177 | 70.0397 | 101.9719 | 115.2836 | 148.5866 |
| Primula macrocalyx | 109.305 | 122.149 | 123.1322 | 101.2671 | 142.8404 | 74.23739 |
| Polygonum hydropiper | 96.42867 | 97.78528 | 102.1904 | 103.6243 | 92.81045 | 88.933 |
| Pimpinella peregrina | 99.93433 | 105.3983 | 114.2642 | 106.6754 | 104.5083 | 116.0622 |
| Mentha longifolia | 133.8927 | 132.7538 | 122.5679 | 113.6243 | 71.22968 | 85.07531 |
| Melandrium album | 78.36144 | 90.95384 | 95.27753 | 78.30183 | 116.2853 | 102.1602 |
| Juniperus oblonga(ST) | 93.64381 | 25.58647 | 118.2297 | 100.7376 | 98.61227 | 97.66548 |
| Juniperus oblonga(LF+FR) | 109.1849 | 126.5164 | 63.36409 | 96.8312 | 97.51782 | 100.4569 |
| Juniperus oblonga(RT) | 103.6064 | 102.4427 | 106.3028 | 130.25 | 133.2536 | 130.3918 |
| Juncus effusus | 103.6718 | 101.898 | 103.0015 | 87.98063 | 95.45552 | 105.1679 |
| Hypericum scabrum | 83.86873 | 29.16687 | 65.01205 | 108.2136 | 64.48058 | 106.2277 |
| Hypericum androsaemum | 113.4267 | 119.1388 | 119.0492 | 137.69 | 140.5197 | 138.9319 |
| Glycyrrhiza glabra | 86.35077 | 89.91277 | 80.76351 | 117.2516 | 126.5483 | 136.1617 |
| Geranium tuberosum | 90.53414 | 100.5426 | 100.3043 | 130.8627 | 117.5391 | 98.57106 |
| Galium verum | 65.42893 | 137.9348 | 126.7347 | 97.31467 | 116.0884 | 118.1276 |
| Eremostachys macrophylla | 79.47345 | 88.63334 | 68.43742 | 113.9539 | 153.7397 | 77.54397 |
| Elaeagnus orientalis | 81.7248 | 82.3261 | 86.49503 | 88.43158 | 85.66378 | 89.55193 |
| Echium russicum | 115.458 | 105.8156 | 101.304 | 108.0994 | 119.1002 | 136.6794 |
| Crataegus orientalis | 106.4726 | 111.139 | 107.4633 | 145.4623 | 136.3497 | 112.7708 |
| Chaerophyllum bulbosum | 90.12625 | 100.2998 | 101.6238 | 94.34245 | 84.90731 | 88.21718 |
| Cardaria boissieri | 78.52831 | 77.27134 | 84.28679 | 96.12005 | 105.0331 | 112.1404 |
| Camphorosma lessingii | 89.81635 | 60.79512 | 65.19455 | 113.0012 | 118.5177 | 115.2464 |
| Caltha polypetala | 109.7567 | 105.8185 | 118.6112 | 74.00206 | 49.72171 | 122.7991 |
| Caccinia macranthera | 104.4214 | 107.7773 | 114.2211 | 140.9494 | 129.0825 | 117.0285 |
| Buxus hyrcana(ST) | 87.74664 | 85.48 | 101.4805 | 95.33321 | 84.46633 | 98.15852 |
| Buxus hyrcana(RT) | 99.50331 | 103.4015 | 96.4177 | 90.96062 | 78.01721 | 54.21484 |
| Astracantha microcephala | 148.2863 | 131.1888 | 130.0841 | 120.2014 | 112.7348 | 129.9648 |
| Atraphaxis spinosa | 123.9683 | 108.0335 | 127.3715 | 145.9729 | 137.7785 | 115.7691 |
| Artocarpus altilis | 90.39783 | 92.87826 | 90.36608 | 102.4372 | 116.9199 | 86.03143 |
| Anchusa azurea | 92.77825 | 101.8936 | 91.12043 | 45.10537 | 53.73939 | 50.78573 |
| Ambrosia artemisiifolia(ST) | 96.13617 | 101.70478 | 100.89155 | 74.35061 | 73.80056 | 79.8878 |
| Ambrosia artemisiifolia(RT) | 149.6594 | 120 | 63.83289 | 106.0169 | 106.6035 | 90.31965 |
| Ambrosia artemisiifolia(LF+FL) | 31.09409 | 100.995 | 97.92355 | 89.50657 | 102.1763 | 89.29678 |
| Achillea biebersteinii | 91.03905 | 69.01466 | 92.33488 | 115.6428 | 134.7719 | 128.353 |
| Prob | 6.532314 | 5.043622 | 4.34122 | 2.375008 | 2.144478 | 0.9311066 |
| Control | 103.3117 | 105.6123 | 91.07597 | 109.5225 | 84.86637 | 105.6111 |

**Figure 3 Raw data**

**OAT1 IC50s**

| **Camphorosma lessingii (D)** | | | |
| --- | --- | --- | --- |
| ug/ml |  |  |  |
| 0 | 98 | 103 | 99 |
| 0.020576 | 89 | 86 | 82 |
| 0.061728 | 88 | 82 | 82 |
| 0.185185 | 66 | 88 | 82 |
| 0.555556 | 64 | 64 | 64 |
| 1.666667 | 46 | 57 | 52 |
| 5 | 37 | 47 | 49 |
| **Geranium tuberosum (B)** | | | |
| ug/ml |  |  |  |
| 0 | 100 | 107 | 94 |
| 0.020576 | 96 | 85 | 87 |
| 0.061728 | 83 | 93 | 91 |
| 0.185185 | 99 | 98 | 91 |
| 0.555556 | 87 | 30 | 88 |
| 1.666667 | 57 | 60 | 53 |
| 5 | 24 | 31 | 29 |
| **Polygonum hydropiper (D)** | | | |
| ug/ml |  |  |  |
| 0 | 100 | 107 | 94 |
| 0.020576 | 81 | 82 | 76 |
| 0.061728 | 71 | 81 | 87 |
| 0.185185 | 84 | 86 | 85 |
| 0.555556 | 49 | 86 | 80 |
| 1.666667 | 50 | 62 | 57 |
| 5 | 39 | 41 | 41 |

**OAT3 IC50s**

| **Anchusa azurea (B)** | | | |  | **Echium russicum(B)** | | | |
| --- | --- | --- | --- | --- | --- | --- | --- | --- |
| ug/ml |  |  |  |  | ug/ml |  |  |  |
| 0 | 98 | 101 | 101 |  | 0 |  | 89 | 111 |
| 0.020576 | 94 | 95 | 103 |  | 0.020576 | 111 | 111 | 114 |
| 0.061728 | 89 | 97 | 95 |  | 0.061728 | 96 | 109 | 101 |
| 0.185185 | 70 | 67 |  |  | 0.185185 | 65 | 62 | 66 |
| 0.555556 | 26 | 33 | 34 |  | 0.555556 | 39 | 37 | 39 |
| 1.666667 | 15 | 15 | 16 |  | 1.666667 | 24 | 22 | 26 |
| 5 | 12 | 11 | 9 |  | 5 | 21 | 20 | 22 |
| **Anchusa azurea (A)** | | | |  | **Glycyrrhiza glabra (D)** | | | |
| ug/ml |  |  |  |  | ug/ml |  |  |  |
| 0 | 98 | 101 | 101 |  | 0 | 97 | 103 | 100 |
| 0.020576 | 93 | 93 | 74 |  | 0.020576 | 87 | 85 | 76 |
| 0.061728 | 103 | 95 | 97 |  | 0.061728 | 83 | 80 | 68 |
| 0.185185 | 94 | 92 | 92 |  | 0.185185 | 86 | 73 | 73 |
| 0.555556 | 97 | 97 | 89 |  | 0.555556 | 82 | 72 | 61 |
| 1.666667 | 69 | 67 | 70 |  | 1.666667 | 66 | 56 | 55 |
| 5 | 36 | 47 | 47 |  | 5 | 41 | 35 | 28 |
| **Astracantha microcephala (D)** | | | |  | **Juncus effusus (D)** | | | |
| ug/ml |  |  |  |  | ug/ml |  |  |  |
| 0 | 96 | 99 | 105 |  | 0 | 102 | 99 | 98 |
| 0.061728 | 89 | 106 | 82 |  | 0.020576 | 98 | 96 | 100 |
| 0.185185 | 79 | 78 | 86 |  | 0.061728 | 91 | 102 | 93 |
| 0.555556 | 81 | 73 | 84 |  | 0.185185 | 81 | 84 | 84 |
| 1.666667 | 70 | 80 | 62 |  | 0.555556 | 65 | 67 | 66 |
| 5 | 43 | 42 | 49 |  | 1.666667 | 45 | 45 | 44 |
| **Chaerophyllum bulbosum (H)** | | | |  | 5 | 21 | 19 | 22 |
| ug/ml |  |  |  |  | **Juniperus oblonga (LF+FR) (D)** | | | |
| 0 | 98 | 102 | 100 |  | ug/ml |  |  |  |
| 0.061728 | 84 | 62 | 90 |  | 0 | 102 | 94 | 104 |
| 0.185185 | 66 | 79 | 80 |  | 0.061728 | 110 | 92 | 93 |
| 0.555556 | 76 | 62 | 80 |  | 0.185185 | 91 | 86 | 75 |
| 1.666667 | 58 | 63 | 66 |  | 0.555556 | 72 | 69 | 74 |
| 5 | 46 | 48 | 47 |  | 1.666667 | 68 | 69 | 64 |
|  |  |  |  |  | 5 | 35 | 39 | 42 |

| **Juniperus oblonga (LF+FR) (B)** | | | | **Primula macrocalyx (D)** | | | |
| --- | --- | --- | --- | --- | --- | --- | --- |
| ug/ml |  |  |  | ug/ml |  |  |  |
| 0 | 103 | 106 | 91 | 0 |  | 89 | 111 |
| 0.06173 | 113 | 110 | 106 | 0.020576 | 104 | 103 | 104 |
| 0.18519 | 105 | 92 | 93 | 0.061728 | 97 | 98 | 99 |
| 0.55556 | 97 | 95 | 87 | 0.185185 | 100 | 100 | 110 |
| 1.66667 | 18 | 13 | 26 | 0.555556 | 82 | 79 | 84 |
| 5 | 16 | 13 | 15 | 1.666667 | 58 | 55 | 60 |
| **Mentha longifolia (B)** | | | | 5 | 24 | 25 | 24 |
| ug/ml |  |  |  | **Symphytum asperum (B)** | | | |
| 0 | 92 | 96 | 112 | ug/ml |  |  |  |
| 0.06173 | 103 | 93 | 88 | 0 | 97 | 103 | 100 |
| 0.18519 | 82 | 97 | 96 | 0.061728 | 77 | 72 | 79 |
| 0.55556 | 60 | 72 | 50 | 0.185185 | 71 | 70 | 67 |
| 1.66667 | 51 | 49 | 58 | 0.555556 | 44 | 44 | 48 |
| 5 | 43 | 44 | 39 | 1.666667 | 22 | 19 | 21 |
|  |  |  |  | 5 | 13 | 14 | 17 |
| **Polygonum hydropiper (D)** | | | |  |  |  |  |
| ug/ml |  |  |  | **Thymus kotschyanus (B)** | | | |
| 0 | 106 | 104 | 90 | ug/ml |  |  |  |
| 0.06173 | 93 | 95 | 92 | 0 |  | 89 | 111 |
| 0.18519 | 73 | 79 | 68 | 0.020576 | 103 | 93 | 98 |
| 0.55556 | 74 | 63 | 64 | 0.061728 | 107 | 62 | 102 |
| 1.66667 | 55 | 51 | 42 | 0.185185 | 81 | 80 | 76 |
| 5 | 24 | 25 | 23 | 0.555556 | 46 | 42 | 39 |
|  |  |  |  | 1.666667 | 25 | 25 | 22 |
|  |  |  |  | 5 | 22 | 24 | 22 |

**Figure 4. raw data**

**Intragastric administration of Juncus effusus**

|  | FS alone | | | | | FS+Juncus effusus (i.g.) | | | | FS+Juncus effusus (i.v.) | | | |
| --- | --- | --- | --- | --- | --- | --- | --- | --- | --- | --- | --- | --- | --- |
| 2 | 85.36172 | 91.84148 | 121.6528 | 181.7607 | 85.36172 | 77.16924 | 93.84062 | 129.8475 | 110.2387 | 108.5121 | 128.3629 | 112.9843 | 90.79917 |
| 5 | 69.51531 | 70.84846 | 112.3429 | 66.57302 | 69.51531 | 69.28088 | 108.7656 | 115.0528 | 111.2579 | 109.5533 | 111.2579 | 106.1909 | 83.64072 |
| 10 | 50.56414 | 61.96338 |  | 54.00262 | 50.56414 | 65.14056 | 93.93877 | 109.6088 | 93.9658 | 104.9608 | 93.62717 | 83.10065 | 73.47762 |
| 15 | 53.5826 |  |  | 47.05024 | 53.5826 | 58.95736 | 87.01329 | 101.0467 | 85.71893 | 89.28091 | 85.71893 |  | 63.57571 |
| 30 | 40.00042 | 41.78153 | 72.78281 | 37.84638 | 40.00042 | 52.5942 | 76.9017 |  | 66.8463 | 57.07581 | 65.91852 | 61.77478 | 46.86834 |
| 60 | 27.72222 | 29.36535 | 50.94242 | 37.19416 | 27.72222 | 53.36213 | 66.87029 |  | 63.62831 | 51.23164 | 69.62831 | 66.10694 | 41.24042 |
| 90 | 22.57045 | 26.17615 | 43.33785 | 30.34287 | 22.57045 | 50.77351 | 54.26349 | 97.61681 | 64.5378 | 44.2303 | 64.62187 | 56.82867 | 34.50827 |
| 120 | 20.05587 | 23.52138 | 24.43171 | 35.69381 | 20.05587 | 50.31121 |  | 66.3354 | 33.08771 | 50.73172 | 33.08771 | 33.24372 | 31.13416 |
